# Cobenefits for Participants of a Nurse‐Led Telephone‐Based Early Childhood Obesity Prevention Intervention: A Multimethod Qualitative Study

**DOI:** 10.1111/ijn.70037

**Published:** 2025-08-10

**Authors:** Sarah Taki, Sarah Marshall, Wendy Smith, Christine Phillis, Annmaree Lavery, Trisha Cant, Jennifer Jones, Paola Gordon, Cathy Llewelyn, Louise A. Baur, Li Ming Wen

**Affiliations:** ^1^ Prevention Research Collaboration, School of Public Health, Faculty of Medicine and Health The University of Sydney Sydney New South Wales Australia; ^2^ NHMRC Centre of Research Excellence in Translating Early Prevention of Obesity in Childhood (EPOCH‐Translate CRE) Sydney New South Wales Australia; ^3^ Health Promotion Unit, Population Health Research & Evaluation Hub Sydney Local Health District Sydney New South Wales Australia; ^4^ Sydney Institute for Women, Children and Their Families, Sydney Local Health District, NSW Health Sydney New South Wales Australia; ^5^ Child and Family Health Nursing, Community Health Services, Sydney Local Health District, NSW Health Sydney New South Wales Australia; ^6^ Susan Wakil School of Nursing & Midwifery, Faculty of Medicine and Health The University of Sydney Sydney New South Wales Australia; ^7^ Discipline of Child and Adolescent Health, Sydney Medical School The University of Sydney Sydney New South Wales Australia

**Keywords:** health promotion, nurses, preventive health programmes, public health, social determinants of health, social support

## Abstract

**Background:**

While preventive health behaviour change interventions target specific behaviours and health‐related outcomes, there can be further benefits, that is, cobenefits, for participants. Healthy Beginnings is an established behavioural intervention targeting mothers of young children to promote optimal child nutrition, physical activity and screen time behaviours and to prevent obesity in early childhood.

**Objective:**

To (1) identify the cobenefits among mothers and children participating in the one‐to‐one telephone support arm of the intervention and (2) explore the factors contributing to these identified cobenefits, both from the perspective of the intervention providers, the Child and Family Health Nurses.

**Methods:**

The telephone‐based Healthy Beginnings intervention was conducted as a randomised controlled trial in NSW, Australia from 2017 to 2019. The intervention, delivered by Child and Family Health Nurses, included nine staged intervention telephone calls from pregnancy to child age 24 months. The nurses' notes from all telephone calls were collated and analysed using content analysis to identify cobenefits. A focus group was conducted with four intervention nurses and analysed using thematic analysis to explore their experiences of delivering the calls and their perceptions of factors that enabled intervention cobenefits for participants.

**Results:**

From the content analysis of the call notes, we derived categories for the types of issues, beyond the target behaviours, for which participants received support. This support primarily pertained to psychosocial and situational factors, for example, relationship challenges. From the thematic analysis of the focus group, we identified two main themes relating to factors that enabled intervention cobenefits for participants: (a) delivery features, relating to the way the intervention was structured and (b) nurse interactions, relating to the way nurses interacted with participants and approached care holistically. The nurses, via the nurse‐initiated staged telephone calls, connected with participants, built rapport, offered tailored child–parent‐centred support and addressed social determinants of health.

**Discussion:**

Scheduled nurse telephone support was crucial for delivering tailored intervention messages for targeted behaviour changes and achieving further cobenefits for participants. Nurse‐led early childhood interventions for optimal nutrition, sleep and movement behaviours have the potential to support families' broader social contextual factors for greater impacts. Behavioural intervention research must capture and consider a broader concept of participant benefits.

## Summary

**What is already known about this topic?:**

○ Health behaviour change interventions are often bound by constraints of clinical trial set‐up (e.g., having one primary outcome), yet such interventions are implemented in a complex system and likely have further outcomes.
○ Participant cobenefits are rarely captured or discussed in health behaviour change intervention trials, and therefore, the effects of the intervention may be underestimated.

**What this paper adds?:**

○ The Healthy Beginnings one‐to‐one telephone‐based intervention delivered by Child and Family Health Nurses offered families holistic support that was broader than the targeted behaviour changes, achieving cobenefits for participants.
○ Key factors that enabled participant cobenefits were the intervention delivery mode and the approach of the intervention providers (i.e., Child and Family Health Nurses).

**The implications of this paper:**

○ Nurse‐led early childhood health interventions for optimal nutrition and movement behaviours have the potential and opportunity to support families' broader social contextual factors for greater impacts.

## 1 Background

Establishing optimal nutrition, movement and sleep behaviours in early life can have lifelong positive health impacts for children, including the prevention of obesity (Mihrshahi and Baur 2018; Woo Baidal et al. 2016). The family unit is central to shaping these early‐life behaviours (Smith et al. 2020a; Australian Institute of Health and Welfare 2021). Interventions aimed at the family level show positive behaviour change effects (Askie et al. 2020; Brown et al. 2019), including those delivered by health professionals (Hennessy et al. 2019). However, family‐focused early‐life behavioural obesity prevention interventions are inherently complex and multifaceted, involving various delivery modes, behaviour change techniques and contextual factors (Seidler et al. 2022; Seidler et al. 2020).

Conventional efficacy studies often adopt a linear ‘pipeline’ approach. Yet multicomponent interventions are multidirectional and are influenced by the complexity of the intervention and the context (Hawe 2015). While the primary outcome serves as the specified ‘endpoint’ for an intervention, the impacts are likely broader than this (Paterson et al. 2009). Less consideration is given to these broader impacts, known as the secondary benefits, or cobenefits, of interventions (Maccagnan et al. 2019). Cobenefits encompass a spectrum of outcomes beyond the primary outcome, such as improved well‐being, education, access to services, social relationships and economic benefits, which can significantly influence participant satisfaction and engagement (Maccagnan et al. 2019; Crisp et al. 2014).

Current evaluation guidance for public health and health promotion interventions directs consideration for contextual factors (Moore et al. 2015), it gives less attention to broader intervention impacts inherent in complex interventions (Skivington et al. 2021; Fletcher et al. 2016). Efficacy studies demonstrate the effectiveness of interventions in early obesity prevention (Askie et al. 2020; Wen et al. 2011; Wen et al. 2022), yet there remains a scarcity of research investigating cobenefits (Crisp et al. 2014), despite their potential significance for understanding intervention effectiveness and broader impacts.

### 1.1 Context and the Current Study

Healthy Beginnings is an established, effective intervention for improving early‐life nutrition and movement behaviours in children (Wen et al. 2011; Wen et al. 2012; Wen et al. 2007). The Communicating Healthy Beginnings Advice by Telephone (CHAT) intervention was conducted as a 3‐arm randomised controlled trial in Sydney, Australia from 2017 to 2019 (Wen et al. 2017). The trial included a control group and two intervention groups: one receiving nurse‐delivered telephone support and the other receiving text message support from late pregnancy until the child was two years old. The predefined primary outcomes were children's objectively measured BMI and BMI *z*‐score at 2 years, and secondary outcomes included child eating, physical activity and screen time behaviours (Wen et al. 2022; Wen et al. 2017; Wen et al. 2020).

Previous evaluation of the trial (Ekambareshwar et al. 2022) examined factors that were associated with higher participant engagement, revealing that participants who scored higher on engagement were reported to have received support on various personal benefits related to psychosocial and social support‐related issues. Further to this, a qualitative study exploring participants' experiences highlighted contextual and psychosocial issues that impacted involvement, including competing issues and emerging health issues (Ekambareshwar et al. 2020). Building upon this evaluation, the current study aimed to (1) identify the cobenefits among mothers and children participating in the one‐to‐one telephone support arm of the intervention from the perspective of the intervention providers, the Child and Family Health Nurses and (2) explore the factors contributing to these identified cobenefits from the perspective of the intervention providers.

## 2 Methods

### 2.1 Study Design

**FIGURE 1:**
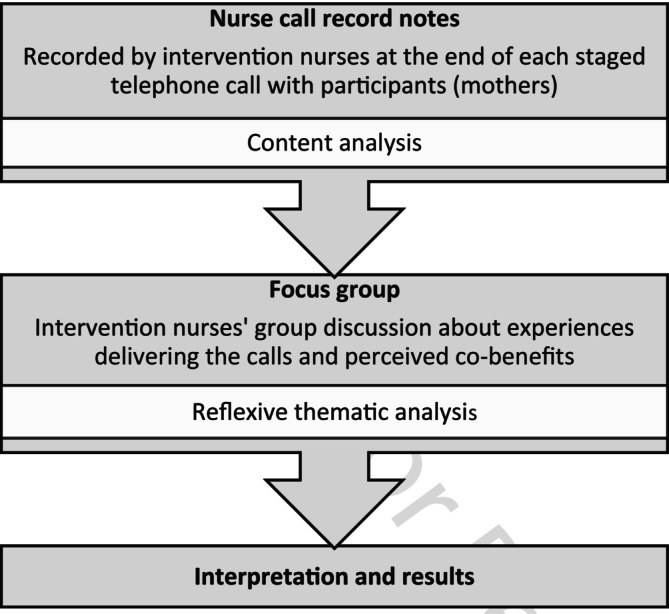
Study design.

This multimethod qualitative study involved a document review of nurses' notes from the participant telephone calls as part of the intervention and a focus group with intervention nurses. The nurses' notes were used to identify participant cobenefits (research aim 1) and a focus group was conducted with the intervention nurses to explore their experiences of delivering the intervention and their perceptions of intervention cobenefits (research aim 2). The data collection methods were sequential and complementary (Figure 1). The research was undertaken from a post‐positivist paradigm. This paradigm is based on the view that what is real is observable, but also acknowledges that this reality is also subjective and there is an interdependence between researcher and subject (Clark 1998).

### 2.2 The Healthy Beginnings Telephone‐Based Support Intervention

The telephone support arm of the Communicating Healthy Beginnings Advice by Telephone intervention involved delivering nurse‐led support at nine stages from late pregnancy to child age 24 months (antenatal, 1, 3, 5, 7, 10 months, 12–15 months, 15–18 months and 18–24 months). These stages align with developmental and nutrition milestones over the first 2 years of life. Each call lasted 30–60 min and followed a predefined outline to ensure coverage of intervention information. Nurses were encouraged to personalise the discussions to address concerns raised by mothers, which included psychosocial support to alleviate mothers' concerns around their health and their children's health and safety.

The telephone intervention providers were experienced Child and Family Health Nurses. In Australia, Child and Family Health Nurses are nurses with postgraduate training that supports families with infants and children aged 0–5 years. Their expertise includes parental and carer physical and mental health, child growth and development, behaviour, feeding and sleep and settling, immunisation and child safety (Schmied et al. 2014). In this study, the intervention nurses received specific additional training to deliver this research programme, including a motivational interviewing training course that supported delivering their practice via telephone. The nurses also received group‐facilitated clinical supervision and support from a Child and Family Health Clinical Nurse Consultant.

### 2.3 Data Collection

#### 2.3.1 Nurse Call Record Notes

The intervention phone calls were conducted from February 2017 to October 2019. Call outlines were structured as data entry tools using REDCap (Research Electronic Data Capture) research management software (Harris et al. 2009) hosted at Sydney Local Health District. Nurses followed call outline prompts and input participant responses accordingly. At the end of each participant's telephone call, the nurses could also record open‐text reflective notes, with the prompt ‘How did you feel about this call? Any additional comments to make?’ These call records were completed directly after each intervention phone call.

#### 2.3.2 Focus Group

In December 2019, following the completion of the intervention, a focus group was conducted with intervention nurses to explore their experiences delivering the calls and their perceptions of participant cobenefits. The focus group method was chosen to create conversations among the purposively selected participants, with their interactions central to generating data (Morgan 1997a).

All four intervention nurses were sent an email invitation to participate, which included the plain language statement and consent form. Voluntary participation was emphasised, and no incentives were offered. Demographic data was not collected to preserve anonymity. The focus group guide, based on the research questions, comprised two main parts: (1) nurses' experiences of delivering the calls and (2) nurses' perceptions of how participants benefited from the programme beyond the intervention target outcomes (see Data S1). The focus group was facilitated by the lead researcher (S.T.) in person at the nurses' workplace, in a private setting without others present.

### 2.4 Data Analysis

#### 2.4.1 Nurse Call Record Notes

A descriptive content analysis (Hsieh and Shannon 2005; Vaismoradi et al. 2013) was undertaken to qualitatively examine nurse call record notes, involving coding and theme identification. This approach was selected due to the nature and quantity of the data; there were over 5000 call records, and the notes were brief and based on objective medical record‐keeping. Following guidelines by Hsieh and Shannon (Hsieh and Shannon 2005) and Elo and Kyngas (Elo and Kyngäs 2008): the analysis began with identifying key words to understand contextual use and then progressed to descriptions and interpretations of the content.

To manage the extensive data, a framework based on potential cobenefits across the social determinants of health (e.g., housing, family socioeconomic status) was developed by co‐authors (S.T., W.S., C.P., A.L. and T.C.) based on a subset of call notes.

After creating definitions for each category, all coders (W.S., C.P., A.L. and T.C.) consistently applied the framework across call record content using a data extraction tool developed in REDCap. Subsequently, a deeper analysis was conducted by open‐coding extracted call record data for each category (S.T., S.M., W.S., C.P., A.L. and T.C.). Summaries were then created based on these codes and discussed before being finalised (S.T. and S.M.).

#### 2.4.2 Focus Group

The focus group transcript was open‐coded and analysed using a reflexive thematic analysis approach, based on Braun and Clarke's six‐stage process (Braun and Clarke 2006). This method was chosen to align with the research objectives and to facilitate a deeper understanding of the data. The stages included familiarisation with the data by reading over the transcript, coding the data, generating themes, reviewing and naming themes, and then finally writing the results. S.T. and S.M. collaboratively undertook the analysis, aiming to build ideas and establish trustworthiness. Themes were developed at a semantic level, where ideas were directly expressed by participants, but also considering underlying, latent ideas. Microsoft Word was used for coding the focus group data and managing the analysis process.

### 2.5 Researcher Roles and Positionality

S.T. and S.M. led the data analysis and interpretation. Both are female post‐doctoral researchers, with training and experience in qualitative methods, who hold tertiary degrees in nutrition and dietetics and doctorates in public health. L.M.W. is a lead investigator for the Healthy Beginnings trial. L.B. has indirectly been involved with the trial, not with project governance or implementation. Co‐authors W.S., C.P., A.L. and T.C. are all female Child and Family Health Nurses and were involved in the CHAT study implementation. They all received qualitative research training and supported guidance from J.J., P.G. and C.L. during the data analysis.

The focus groups were facilitated by S.T. S.T. and the participants had worked together on the trial for 2 years before the commencement of this substudy. The nurse participants knew the reasons for undertaking this study and had a shared interest in answering the research questions. While pre‐existing relationships between the facilitator and participants are sometimes viewed as a limitation (Morgan 1997b), in this instance, they were seen as advantageous due to the intimate knowledge of the intervention and the strong working relationships that fostered open communication.

### 2.6 Ethical Approval and Informed Consent

This research was granted ethics approval from the Ethics Review Committee of Sydney Local Health District (Protocol No: X16‐0360 & LNR/16/RPAH/495 and Protocol No X18‐0387 & HREC/18/RPAH/545). All participants received information about the study, had opportunities to ask questions about the research, and subsequently gave their consent to participate. All data and quotes were anonymised.

## 3 Results

### 3.1 Identifying Participant Cobenefits

Nurse call notes were analysed from nine intervention time points for 386 mothers (primiparous and multiparous) allocated to the telephone intervention at baseline (Ekambareshwar et al. 2018). At 2 years, 246 mothers completed follow‐up (64% retention); further details regarding the sample and engagement are provided elsewhere (Wen et al. 2022).

The content analysis of nurse call record notes revealed mothers' discussions of key challenges extending beyond the intervention's focus. Table 1 describes the 10 domains and the subthemes derived. Mothers sought and received support from nurses on various issues, including relationship challenges, financial stress and mental health concerns. Intervention nurses commonly made referrals (with participant consent) to Child and Family Health services, external services (e.g., domestic violence support or perinatal depression counselling) and online resources (such as government websites like Raising Children Network).

**TABLE 1:** Content analysis of nurse intervention call notes to identify challenges that mothers discussed beyond intervention focus.

Key categories	Themes derived and description
**Family**	
Domestic abuse	*Disclosure of previous or current abuse experienced*. Mothers who chose to disclose described different forms of abuse such as financial, emotional, and/or physical abuse. This may have occurred previously but continued to impact them or was occurring in the present. *Lack of support and/or knowledge to change situation*. Often mothers who disclosed current abusive situations described uncertainty about how to improve their situation. *Support referrals to relevant services*. Nurses guided mothers to appropriate services encouraging them to make self‐referrals, or in more extreme cases made referrals directly to appropriate services with mothers' consent.
Partner	*Relationship and parenting tensions*. Some mothers described challenges of having differing contributions and views to their partner toward parenting and care for their child. This often resulted in relationship pressures. Other sources of relationship strains included partner relationship changes, conflicts, partner's wellbeing, and employment situation.
Family relationships	*Family dynamics*. At times mothers discussed relationship challenges with family members, such as maternal or paternal grandparents or siblings, or the child's siblings. This often influenced the child's care and the mother's wellbeing. *Family support with caring for the child*. Mothers had mixed experiences with supports provided by family. The key challenge expressed was family not living in the same area/city and so unable to help. *Family health concerns*. For some mothers, they were also concerned with and supporting family members with disability or illness. Often, this added great strain for these mothers.
**Resources**	
Housing	*Moving houses*. This often brought about stress for mothers. Reasons for moving were varied, commonly to be closer to family and friends, but also included relationship breakdown and employment changes. *Troubles with housing and/or living situation*. Some mothers expressed feeling stressed with living arrangements, e.g., living with extended family or in small living areas or struggling with hardships, e.g., unable to make housing payments and living in a refuge.
Financial stress	*Family employment circumstances and financial assistance*. Unemployment, unstable work, and single incomes were challenges for some families. At times, mothers talked about Centrelink or work compensations for assistance. *Financial situation implications*. Some mothers talked about issues arising because of their financial hardship, such as needing to move house, borrow money, or not being able to afford items such as fresh foods or baby equipment. Some mothers described feeling guilty, anxious and worried because of their financial stress.
**Personal**	
Migration, culture and language	*Resources for different languages*. Mothers from language backgrounds other than English were offered telephone interpreters. AT times language was a barrier. Some mothers requested language‐specific written materials and local playgroups for social connections. *Cultural practices*. At times mothers described cultural practices from their country of origin that were different to those in Australia, such as confinement (post‐natal recovery period), use of specific foods for wellbeing, and traditions of early introduction of solid foods. *Varied support networks*. Some migrant mothers described a lack of family support, family living overseas, feelings of homesickness, and/or social isolation. Some described having family longer‐stay visitors and/or regular video calls with family overseas.
Work and study	*Challenges of returning to work*. Many mothers mentioned feeling anxiety about returning to work, describing reasons such as work stress, separation from their child, juggling time and priorities, concerns about cost and timing of childcare. Conversely, some mothers spoke of mixed feelings or feeling happy to return to work. Challenges of breastfeeding and returning to work were also raised. *Varied work and study situations*. Mothers shared their diverse work and study situations with the nurses, e.g., work/study hours per week, childcare arrangements and job changes. Work stress was a common issue among mothers who had returned to work.
Mental health	*Emotional changes and postnatal depression*. Mothers expressed feelings of low mood, mood swings, anxiety and depression. Mothers described a range of contributing factors to this, e.g., history of mental illness such as postnatal depression, feeling generally worried or overwhelmed, situations such as birth trauma, illness, grief and loss, or relationship stress. At times, mothers talked through complex and ongoing situations with the intervention nurses. *Social isolation and loneliness*. Social isolation and loneliness were more common among mothers without family nearby. Some mothers also described changes in friendships after having a child and feeling isolated at home caring for their child. *Mental health support and community connections*. At times, mothers had professional support from psychologists or counsellors and/or prescription medication, yet some mothers were not accessing any services. As appropriate, the intervention nurses often encouraged self‐referrals to support services such as local general practice doctors and the Perinatal Anxiety & Depression Australia (PANDA) free national helpline. The intervention nurses also encouraged mothers to join local parenting groups and playgroups for social connections.
**Medical**	
Mothers' medical issues	*Information and support for maternal health issues*. Mothers raised a range of their own health concerns, particularly medical issues related to pregnancy, childbirth and postpartum, e.g., gestational diabetes, pelvic pain, incontinence and prolapse. The intervention nurses were able to provide direct support over the phone and/or refer mothers to local doctors or specific to services.
Children's health issues	*Information and support for general child health issues*. Mothers raised a range of child health concerns as relevant to them, and nurses were able to provide direct support over the phone and/or refer families to services for assessments. For example, common general health issues included skin rashes, infections, reflux, constipation, speech and developmental delays, tongue tie.

### 3.2 Exploring Factors Influencing Participant Cobenefits

**FIGURE 2:**
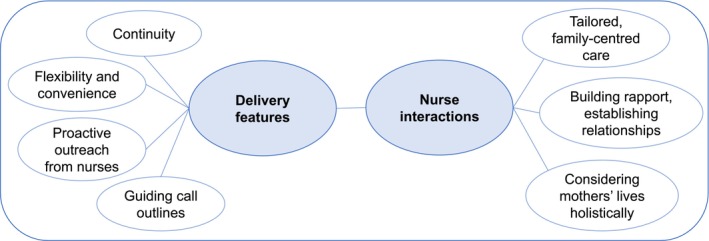
Thematic map of factors enabling participant cobenefits.

The focus group analysis explored the intervention nurses' experiences with delivering the calls. Two interconnected themes were identified: (1) delivery features and (2) nurse interactions, both contributing to the overarching concept of building trust and support. The subthemes specify the key factors that enabled the cobenefits. Figure two illustrates the thematic map (Figure 2).

#### 3.2.1 Theme 1: Delivery Features

The theme ‘delivery feature’ encompasses both intervention characteristics and factors related to the setup of the intervention, with four subthemes: 1.1 continuity, 1.2 flexibility and convenience, 1.3 proactive outreach from nurses and 1.4 guiding call outlines.

The subtheme ‘continuity’ (1.1) pertains to the sense of ongoing connection mothers felt as participants in the Healthy Beginnings programme, characterised by consistent communication with the same nurses over the programme's duration (from 0 to 2 years). This continuity fostered trust between families and the nurses. One nurse reflected on this: ‘I think the fact that there was only a small number of us [intervention nurses] that [mothers] often spoke to us a few times, so they got that continuity’ *‐ Nurse 3*.

The intervention nurses highlighted ‘flexibility and convenience’ (subtheme 1.2) as a key aspect of the programme setup, benefiting families. Flexibility in call timing and scheduling accommodated families' needs, including after‐work hours or weekends. Nurses were available for longer calls if necessary. Phone delivery was convenient for mothers, allowing them to receive support from home, particularly beneficial for families living in rural or regional areas. The nurses supported this: ‘It's just a whole new level I think, rather than being in a clinic where you've got your 45 minutes …, [instead,] having that ability to really listen to them [mothers] and really get to know some of them. It's been fantastic’ *‐ Nurse 4*.

The subtheme ‘proactive outreach from nurses’ (1.3) underscores the nurse‐initiated nature of the calls, which were staged as per intervention procedures. Additionally, mothers had the option to contact the nurses if any issues arose. Nurses emphasised the significance of this proactive approach, especially for mothers who were not initiating appointments with universal child and family nurse services or family doctors. One nurse describes this: ‘There's a bit of work going on about how beneficial it is to have a nurse‐initiated call, rather than waiting for women to [initiate] … we were able to call them to see how things were and were able to make a good clinical judgment.’ *‐ Nurse 2*. Another nurse echoed this sentiment: ‘But then the positive to that is being able to call them [mothers] and just catching them if they did pick up. They might have been having a really bad day, and then they just disclose to you what's really going on for them, and that turned into a positive because you're able to support, advise, refer, et cetera’ *‐ Nurse 4*.

The last subtheme, ‘guiding call outlines’ (1.4), relates to the value of the intervention call outlines as a guide for covering intervention content during the scheduled calls with mothers. These call outlines allowed for tailoring for each mother and prompted mothers to talk about specific topics that get little coverage in universal well‐child appointments, such as mothers' well‐being and goal setting for behaviour changes. This intervention characteristic was an important feature in shaping nurse‐participant interactions, as described in Theme 2. One nurse described this: ‘We've got the script [call outline], we have the health outcome, and we're really thorough about addressing each of one of those. Whereas if you had your appointment with them, you'd have to address the things that are most important and try to then get through the other stuff’ *‐ Nurse 2*.

#### 3.2.2 Theme 2: Nurse Interactions

The theme ‘nurse interaction’ encompasses the intervention providers' (nurses') interactions with participants, which played a crucial role in achieving programme outcomes with mothers, including both planned and unplanned intervention benefits. This theme includes three interrelated subthemes essential to the nurses' successes: 2.1 Tailored, family‐centred care, 2.2 Building rapport and establishing relationships, and 2.3 Considering mothers holistically.

The subtheme ‘tailored, family‐centred care’ (2.1) refers to the nurses' approach to providing care for the participating families. Nurses described addressing mothers' questions and responding to their needs, helping them to problem solve, set goals and apply the intervention key messages tailored to each individual's circumstances. The nurses saw this as essential to providing personalised, person‐centred care. In discussing the tailored care, one nurse described the challenges in negotiating the system: ‘It's hard. The women don't know what the services are. We don't know what the services are. We can find out because we've got the health literacy and we can be their advocate, and that's what we did time and time again, was to get the support that they needed’ *‐ Nurse 2*. Another nurse also commented on the frequency of individualised referrals: ‘[I am] definitely referring to different services that I'd never referred to in my job in a clinic’ *‐ Nurse 4*.

The subtheme ‘building rapport and establishing a relationship’ (2.2) describes the nurses' efforts in establishing and nurturing relationships with participating families. This approach is critical in nursing practice, but also something the nurses felt that they were enabled to do given the long intervention period (2 + years) (linking to subtheme 1.1 continuity). One nurse described this: ‘You develop this rapport. You've got to know these families, you've travelled with them, and some of them have really taken on everything we've said. So, it's been amazing that we've had that sort of an impact. I think that's always surprised me, that we've always had such a positive impact’ *‐ Nurse 4*. Another nurse expressed a level of surprise about the ability to build such relationships over the phone: ‘It's been interesting to see how open they are, and the things that they tell us over the phone, having never met us, and I guess, the rapport building and how interesting that's been’ *‐ Nurse 1*.

The last subtheme (2.3), ‘considering mothers’ lives holistically,’ encapsulates the nurses' awareness and responsiveness to the broader context of mothers' lives during their interactions. Beyond solely delivering intervention messages, nurses recognised and addressed other higher‐priority issues that families faced. The nurses understood the need to consider mothers' broader concerns, to then be able to assist with target behaviour changes. One nurse put it directly: ‘Well, obesity prevention is all very well and good, but if you're a woman that is suffering from depression, you are probably not going to be worried about … five serves of vegetables a day, you know, that sort of thing. So, you need to look holistically at a person rather than a single part of them’ *‐ Nurse 1*.

## 4 Discussion

This study explored participant cobenefits from the telephone support arm of the Communicating Healthy Beginnings by Telephone intervention, along with factors that contributed to these cobenefits. The identified cobenefits encompassed factors related to the social determinants of health, including psychosocial support for financial security, relationship challenges and mental health. The intervention delivery characteristics and intervention provider (Child and Family Health Nurse) approaches were central to achieving participant cobenefits. This research enhances our understanding of the impacts of the telephone support intervention and, more broadly, offers insights into the evaluation of cobenefits within complex interventions.

The Communicating Healthy Beginnings Advice by Telephone intervention successfully influenced specific target behaviours at child age 2 years (i.e., no bottle use at bedtime, family mealtimes, drinking from a cup, less screen time, and not watching television at dinner time); however, it did not significantly impact child BMI, the primary outcome measure (Wen et al. 2022; Wen et al. 2020). This current study highlights that behaviour change interventions can yield benefits beyond predefined primary and secondary outcomes and underscores the significance of considering broader impacts, such as psychosocial support and referral pathways, in intervention design and evaluation.

### 4.1 Addressing a Gap in the Literature

Participant cobenefits remain largely unexplored in early‐life behavioural interventions for obesity prevention. Although some insights may emerge during qualitative evaluations, such as in the case of a behavioural sleep intervention where parents identified cobenefits (Tse and Hall 2008), we did not find planned data capture or substudies specifically focusing on cobenefits. However, measuring cobenefits is crucial and holds the potential to enhance our understanding of early‐life behavioural interventions.

A 2023 systematic review by Brown and colleagues examined the broader impacts, or spillover effects beyond participants, of childhood obesity prevention interventions (Brown et al. 2024), finding that positive effects can be observed among parents and families of targeted participants. This finding is complementary to our study, highlighting the complexity of behavioural interventions and the potential for positive health impacts for individuals participating in the intervention (cobenefits) and for populations proximal to the target population (spillover effects). Measuring and considering benefits beyond intervention target behaviours and target populations adds to our understanding of overall intervention effects, which is increasingly important in real‐world implementation and scale‐up.

Assessing and acknowledging benefits beyond targeted behaviours and populations contribute to our comprehension of overall intervention effects, which is increasingly vital for real‐world implementation and scale‐up efforts.

### 4.2 Importance of Addressing Social Determinants to Enable Behaviour Change

Individual health and well‐being are greatly influenced by contextual factors, particularly personal circumstances and community environments (World Health Organization 2023). Despite extensive research, known risk factors such as breastfeeding duration, movement practices and sleep behaviours only explain a portion of obesity risk (Mihrshahi et al. 2018). Research indicates that these risk factors are shaped by contextual factors such as mental health, social support and financial support (Pezley et al. 2022). For example, the availability of social support during the postnatal period can positively impact breastfeeding duration (Page et al. 2022; Chambers et al. 2023). These study results highlight the importance of the Healthy Beginnings intervention nurses prioritising such social support, for example, identifying and supporting families with child protection issues in domestic violence situations or with extreme financial difficulties. Addressing social determinants of health (such as income, education and environment) can also influence participant satisfaction and engagement with a behavioural intervention (Maccagnan et al. 2019; Crisp et al. 2014). We found that addressing social determinants with participants fostered trust and support from the nurses' perspectives. This can enhance participant satisfaction and acceptability of the intervention, which in turn can support target behaviour change. The mechanisms of action warrant exploration. Social determinants of health, beyond individual behaviours, are crucial in shaping overall intervention health outcomes.

### 4.3 Intervention Providers as Enablers of Intervention Cobenefits

Our findings highlight their unique contribution of the intervention providers, the Child and Family Health Nurses, in achieving the targeted outcomes and intervention cobenefits for participating families. This is partly related to the unique profession of Child and Family Health nursing that focuses on optimal child growth and development in the first 5 years of life (Wightman et al. 2022). In the past decade, these nursing practices have evolved to emphasise working in partnership with parents, supporting parent decision‐making (Wightman et al. 2022; Fowler et al. 2012). Addressing child growth and health behaviours related to nutrition, sleep and activity is recognised by Child and Family Health Nurses as part of core business (Cheng et al. 2020), and their practice of working in partnership with families enabled the nurses in the Healthy Beginnings trial to comprehensively support mothers with social factors and also targeted behaviours. Unlike other health professions, such as doctors or practice nurses, Child and Family Health Nurses possess specialised training and a focus on the social determinants of health, allowing for more holistic and supportive care. Additionally, our study revealed that intervention nurses adopted a flexible approach, allowing mothers to guide the conversation, which aligns with research describing parents' preferences for practical, evidence‐based and non‐judgmental support in healthy child growth interventions (Hennessy et al. 2020) and breastfeeding support (McLardie‐Hore et al. 2022).

### 4.4 Intervention Characteristics as Enablers of Intervention Cobenefits

An important element of the telephone‐based Healthy Beginnings trial was the delivery of the intervention by Child and Family Health Nurses via telephone. While previous research, such as a 2013 Cochrane review, revealed somewhat promising yet inconclusive results regarding telephone‐based maternity services (Lavender et al. 2013), the past decade has seen an increase in the utilisation of telephone delivery and online technologies, particularly with the onset of the COVID‐19 pandemic (Smith et al. 2020b; Jaynes et al. 2022). In this study, using telephone as the mode of delivery allowed flexibility for nurses and mothers. For the nurses, it allowed the calls to be conversational and adapted to cater for the mothers' needs. For mothers, it facilitated engagement with those living in regional areas and those with limited English proficiency (as a telephone interpreter was arranged by the nurses), and also provided flexibility for parents to schedule calls at convenient times. The employment of dedicated Child and Family Health Nurses for the entirety of the intervention duration ensured consistency and continuity of care for participating mothers. Continuity of providers is a well‐established factor for quality and acceptable healthcare (van Walraven et al. 2010). A final key element of the intervention delivery was the proactive outreach from the intervention nurses aligned with stages of child development. This outreach strategy enhanced accessibility and engagement in the intervention, overcoming barriers for parents such as uncertainty about available services or how to access them (Rossiter et al. 2019). While not identified in the analysis, it is possible too that the perceived anonymity of a phone call as opposed to a face‐to‐face consultation may have encouraged the parent to express concerns more easily, resulting in the parent cobenefits.

### 4.5 Study Implications

#### 4.5.1 Developing a Conceptual Model

**FIGURE 3:**
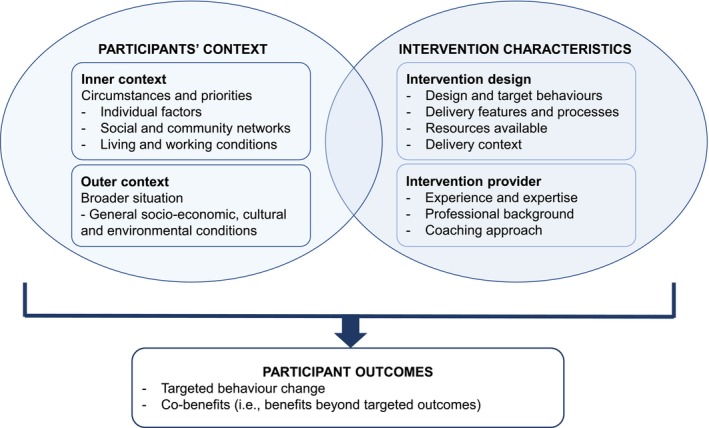
Conceptual model of participant context and intervention characteristics in achieving participant outcomes of provider‐delivered behaviour change interventions.

Based on our findings, we developed a conceptual model (Figure 3) illustrating the connection between the intervention factors that promoted the cobenefits and the resultant participant cobenefits. There are important interactions between the intervention (design and provider) and the participants' inner and outer context (inner context being individual or immediate level factors, and outer context being broader societal and environmental factors). These interactions are shaped by the distinctive attributes of each factor, influencing the attainment of targeted and secondary intervention benefits (cobenefits) for participants. While the conceptual model does not delve into the mechanisms of behaviour change, it highlights the critical importance of considering participant context in understanding and mapping intervention outcomes (both planned and additional cobenefits). Notably, this model is tailored to provider‐delivered behaviour‐change interventions, where the intervention provider plays a pivotal role in achieving desired outcomes.

#### 4.5.2 Considerations for Future Research

Our study highlights broader positive outcomes alongside proposed intervention goals and the complexities involved in behaviour change interventions, underscoring the need for further research in this area. We urge interventionists to explore methods of capturing participant cobenefits and consider the potential impact of cobenefits on participant engagement and satisfaction, targeted intervention outcomes, and implementation efforts. Incorporating participatory and co‐design approaches may offer potential avenues to address the complexity of social factors influencing health when designing interventions (Rossiter et al. 2019). Our research also highlights the importance for interventionists to consider integrating the social determinants of health with the science of behaviour change when designing and delivering interventions (Schulz et al. 2002).

### 4.6 Limitations

This study leveraged existing data collected as part of the intervention (nurses' call record notes) to opportunistically identify the participant cobenefits. Because of this, the participant cobenefits are from the nurse perspective and may not cover all cobenefits. This study followed on from an evaluation involving the participating mothers (Ekambareshwar et al. 2022), so we were unable to engage the mothers again for this study. A key recommendation for researchers is to intentionally consider collecting, analysing and reporting on intervention cobenefits. Furthermore, our findings may be specific to interventions with similar delivery characteristics and intervention providers, potentially limiting the generalisability of the results. Nonetheless, the study offers valuable insights for researchers and policymakers, emphasising the importance of comprehensive prevention‐based health promotion programmes that address broader factors, such as social determinants of health, beyond targeted behaviours.

## 5 Conclusions

Proactive outreach telephone support from Child and Family Health Nurses was crucial, not only for delivering tailored Healthy Beginnings intervention messages but also for achieving participant cobenefits beyond the targeted behaviours, particularly psychosocial support. The intervention delivery characteristics and intervention provider approaches were pivotal in achieving cobenefits. Nurse‐led early childhood interventions for optimal nutrition, sleep and movement behaviours have the potential to support families' broader social contextual factors for greater impacts. Our findings emphasise the importance of considering cobenefits in behavioural intervention research and expanding our perspectives on how participants may derive benefits from such interventions, extending beyond primary and secondary health outcomes.

## Author Contributions

**S.T.:** conceptualisation, project administration, data curation, methodology, investigation, formal analysis, visualisation, supervision, writing – original draft, writing – review and editing. **S.M.:** conceptualisation, data curation, methodology, formal analysis, visualisation, writing – original draft, writing – review and editing. **W.S., C.P., A.L. and T.C.:** investigation, formal analysis, writing – review and editing. **J.J., P.G. and C.L.:** supervision, writing – review and editing. **L.M.W. and L.A.B.:** conceptualisation, funding acquisition, resources, supervision, writing – review and editing.

## Disclosure

The authors declare that the funders (NSW Health and NHMRC) played no role in the design and conduct of the study; collection, management, analysis and interpretation of the data; preparation, review or approval of the manuscript; and decision to submit the manuscript for publication.

## Conflicts of Interest

The authors declare no conflicts of interest.

## Supporting information


**Data S1:** Supporting Information.

## Data Availability

The raw data from this study will not be shared to ensure the protection of participants' privacy and confidentiality. Additionally, the data contain information that could be identifiable, so due to the sensitive nature of the research, supporting data are not available.
