# Supplementary material for: Cobenefits for Participants of a Nurse‐Led Telephone‐Based Early Childhood Obesity Prevention Intervention: A Multimethod Qualitative Study
Source: Int J Nurs Pract. 2025 Aug 10;31(4):e70037. doi: 10.1111/ijn.70037 (PMC12336438; doi:10.1111/ijn.70037)
Supplement: Supplementary file 1 — Data S1: Supporting Information. [file IJN-31-e70037-s001.docx]

# Supplementary File 1 - **Focus group guide**

The purpose of this session is to talk about your experiences with delivering the Communicating Healthy Beginnings Advice by Telephone (CHAT) Study. More specifically, today we will focus on your experiences with delivering the telephone calls and the unanticipated outcomes.

**General experience delivering the study intervention**

1. To start off with, can you express your overall experiences with delivering the Healthy Beginnings CHAT study, in particular your experiences with delivering the telephone support program?

**Barriers and facilitators delivering the telephone intervention**

*As you are aware the main outcome of the CHAT study was to encourage healthy weight gain in the first few years of life for the prevention of overweight and obesity in children. The key messages associated with addressing obesity prevention that you addressed in the study included infant feeding behaviours, active play and sleep in children and mothers’ well-being.*

1. What do you feel worked well when you delivering this advice to mothers?
   1. What do you feel motivated mothers to adopt these behaviours?
2. Were there any topics that most mothers sought your advice about?
3. Were there topics you found challenging to deliver over the phone?
4. What about with mothers, were there any topics you felt was challenging for mothers to understand or practice?
5. In your opinion, what factors impacted on the delivery of the Healthy Beginnings telephone calls to mothers?
   1. What were the critical success factors?
   2. What were the critical barriers?

**The unanticipated outcomes delivering the telephone intervention**

*Although the main outcome of this study focused on addressing obesity prevention and the related behaviours that I mentioned earlier, one of the aims of this focus group is to discuss the unanticipated outcomes that you may have experienced while you were delivering these telephone calls with mothers.*

1. Can you tell me more about what these unanticipated outcomes may have included?

*Prompt: education, goal setting, emotional and social support, referrals to other professionals, targeted hard to reach populations*

1. For a study that is focused on obesity prevention, how important do you feel is it to address/acknowledge these unanticipated outcomes?
2. What implications if any, do you feel these unanticipated outcomes have on the study outcomes that are focused on behaviour change or the uptake of healthy behaviours?
